# Adipogenic Effect of Magnolol in Primary Human Pre‐Adipocytes With Potential Skin Health and Volumizing Effect

**DOI:** 10.1111/jocd.70066

**Published:** 2025-03-26

**Authors:** Alan D. Widgerow, Nava Dayan, Mary E. Ziegler, Faiza Shafiq

**Affiliations:** ^1^ Division Chief Research, Professor Plastic Surgery, Center for Tissue Engineering University of California Irvine California USA; ^2^ Alastin Skincare, Inc A Galderma Company Fort Worth Texas USA; ^3^ Nava Dayan Ltd Shoham Israel

**Keywords:** adipogenesis, adiponectin, CCAAT/enhancer‐binding protein alpha (c/EBPα), fatty acid binding protein 4 (FABP4), magnolol (ML), perilipin (PLIN), peroxisome proliferator‐activated receptor (PPARγ), subcutaneous white adipose tissue (sWAT)

## Abstract

**Background:**

Aging is associated with fat atrophy and fibrosis with loss of adipocyte differentiation from preadipocytes. New approaches to this loss involve agents that can renew the proliferative and differentiative capacities of preadipocytes with the aim of creating new healthy adipose tissue that secrete adipokines that positively impact on skin health.

**Material & Methods:**

We investigated the effect of Magnolol (ML), a naturally derived compound, on human primary pre‐adipocyte viability and proliferation as well as adipogenic gene expression and increase in lipid production. Cell proliferation was assessed using fluorescent signaling, and adipocyte differentiation was monitored by following morphological and microscopic changes. RNA purification and real‐time PCR were undertaken to examine gene expression changes, and Oil red O staining was used to confirm adipose cell transformation. Adipokine expression, in particular adiponectin quantification, was also undertaken.

**Results:**

Magnolol, at a relatively low concentration, demonstrated clear adipogenic activity: with a significant increase in preadipocyte proliferation after 48 h and a significant accumulation of adipocytes as demonstrated by oil red staining. Increased gene expression of PLN1 and FABP4 and a significant increase in adiponectin protein expression was demonstrated.

**Conclusion:**

Magnolol stimulates preadipocyte proliferation and conversion to adipokine‐producing adipocytes. This has the potential for a positive skin health and volumizing effect if used in a topical formulation.

## 1 Introduction

Skin health is influenced by multiple factors including level of moisture, the integrity and structure of the extracellular dermal matrix as well as by fat content and distribution. Among these factors, fat as a component is the least studied. Facial structural changes in aging are associated with volumetric loss of fat, bone resorption and redistribution of soft tissue [1]. The subcutaneous white adipose tissue (sWAT) is the predominant type of fat in the human body. Below the dermis it is termed “subcutaneous fat”. sWAT serves as the reservoir for energy storage in the body and is a source of multiple lipids and protein factors involved in functional regulation. It has been established that both the rate of adipogenesis, that is, the accumulation of adipocytes in the tissue, and the replication and differentiation of preadipocytes, are reduced in older subjects [2, 3].

Dermal white adipose tissue (dWAT) represents a specialized fat depot present in the deep dermis commonly situated at the base of the hair follicle that can undergo phenotypic changes in aged, photodamaged skin. A reduction of the dWAT volume and fibrotic replacement of adipocytes is evident in photodamaged skin [4]. For this reason, researchers have also identified dermal adipocytes as targets in skin anti‐aging strategies [4].

Magnolol (ML) is a polyphenolic component that exists (together with its isomer honokiol) in the plant magnolia officinalis. It has been reported to decrease blood glucose and insulin levels in type 2 diabetes model rats and importantly from the perspective of this study, to induce the differentiation of murine preadipocyte cells into adipocytes [5].

The objective of this study was to evaluate the effect of ML as an adipogenic agent. ML was derived and purified from magnolia bark (Orient Stars LLC. Gardena CA, USA) Its effect on human primary pre‐adipocytes was assessed and its potential mode of action was investigated via gene and protein expression, and cell morphology observations. This is intended to validate the inclusion of ML in a topical formulation as a volumizing or fat tissue modulating agent, recognizing that changing the nature of aged fatty tissue to more healthy fatty tissue has positive skin health effects.

## 2 Materials and Methods

### 2.1 Cell Culture

Normal human primary subcutaneous pre‐adipocytes (Cat #: PCS‐210‐010, ATCC Manassas VA) were obtained from 33‐year‐old healthy Caucasian woman and cultured according to manufacturer's instruction. Cells were seeded at a density of 5000 cells/cm^2^ with complete medium: Fibroblast basal medium (Cat #: PCS‐201‐030, ATCC, Manassas, VA, USA.) supplemented with Fibroblast growth kit‐Low serum (Cat #: PCS‐201‐041, ATCC) and a Penicillin–Streptomycin (P/S) solution (Cat #: 03–031‐1B, Sartorius, Gottingen, Germany) (Penicillin: 10 U/mL, Streptomycin: 10 μg/mL). Medium was exchanged 24 h after seeding and then every 2–3 days, until the monolayer reached a 70%–80% confluency. For sub‐culturing, the cells were incubated with trypsin (Cat #: 03–053‐1A, Sartorius) and neutralized with an equal volume of FBS. Before seeding cells on the plate, viability and cell number were evaluated by Trypan Blue exclusion assay. A viability of above 85% was considered acceptable. The cells were maintained in a humidified 5% CO_2_ atmosphere at 37°C.

### 2.2 Cell Viability and Proliferation

Cell viability was evaluated 24 and 48 h after treatment with Magnolol 31.6 μM, using the WST‐1 reagent (Cat #: 5015944001, Merck, Darmstadt, Germany), following the manufacturer's instructions. Six replicates were used for each section of testing. The absorbance was measured at 450 nm after 1.5–3 h of incubation using a BMG Labtech CLARIOstar plate‐reader (BMG Labtech, Germany). The values are expressed relative to untreated cells. The concentration of ML used was determined to be noncytotoxic and nonantiproliferative at previous experiment conducted (not shown).

### 2.3 Differentiation Procedure

Cells were seeded (day 0) at a density of 18 000 cells/cm^2^ and incubated with complete medium for 48 h before initiating pre‐adipocytes differentiation. Differentiation was triggered by either ML (Cat #: CH0630, Orient Stars LLC, CA, USA) or an Adipocyte Differentiation Toolkit, which served as a control (Cat #: PCS‐500‐050, ATCC). ML was dissolved in DMSO to prepare a 50 mM high‐concentration stock solution, which was stored at −20°C. Before use, it was diluted in complete medium to its final concentration. For the ML treatment, DMSO was used at a final concentration of 0.062%.

Differentiation course: After 48 h (day 2), cells were washed with PBS and culture medium was substituted with complete medium supplemented with 3.1 μM ML or with differentiation medium. Then, every 48–72 h (days 7, 9, 11, 14, 16, 18), 66% of the culture medium was substituted with either fresh complete medium supplemented with 3.1 μM ML or with maintenance differentiation medium (Adipocyte basal medium enriched with ADM Supplement, Cat #: PCS‐999‐042, ATCC). Adipocyte differentiation was monitored by following morphological and microscopic changes.

### 2.4 RNA Purification

Total RNA was extracted on days 8, 11, 15 and 18 of the differentiation, as described above. The cells were lysed with TRI reagent (Cat #: R2050‐1‐50, Zymo research, Irvine, CA, USA). RNA was extracted by using Direct‐zol RNA MicroPrep (Zymo Research) (50 Preps) w/ Zymo‐Spin IC Columns (Capped) (Cat #: R2061‐A, Zymo research) following the manufacturer's instructions. A Denovix DS‐11 FX (Wilmington, DE, USA) was used for RNA quantification. Purified RNA was stored at −80°C.

### 2.5 Real‐Time PCR

cDNA was synthesized using a High‐Capacity cDNA RT kit with an RNAse inhibitor (Cat #: AB‐4374966, Thermo Fisher Scientific, Waltham, Massachusetts) using 0.48 μg of total RNA per 20 μL reaction, using a Blue Ray Biotech Turbocycler. cDNA was stored at −20°C. Real‐time PCR was performed on the Bio‐Rad CFX96 Real‐Time PCR system C1000 touch, using the PowerTrack SYBR Green Master Mix (Cat #: AB‐A46109, Rhenium) according to manufacturer's instructions. The primer sets used in this study were costume made by Integrated DNA Technologies (IDT) and used at a final concentration of 0.3 mM each (Table S1). PCR amplifications were performed under the following conditions: (1) 2 min at 95°C, (2) followed by 40 cycles of 5 s at 95°C and 30 s at 56°C and (3) post amplification melting curve: 15 s at 95°C (1.99°C/s), 1 min at 52°C (1.77°C/s) and 15 s at 95°C (0.075°C/s).

### 2.6 Oil Red O Staining

The differentiation procedure was performed for 18 days as detailed above. The Oil Red assay kit (Cat #: MAK‐194, Sigma‐Aldrich) was used according to manufacturer's instructions. Counterstain of the nuclei was performed using hematoxylin. The cells were photographed at 10x and 20x magnification with an Olympus inverted microscope. Images were analyzed by ImageJ software (Manufacturer and location) The percentage of area occupied by Oil Red O stain was calculated.

### 2.7 Adiponectin Quantification in the Cell Culture Supernatant

The differentiation procedure was performed as detailed above. On days 8, 11, 15 and 18, 0.5 mL of cell culture media was collected from the wells, and the adiponectin concentration was determined using a Human Adiponectin ELISA kit (Cat #: ab‐99 968, abcam, Cambridge, UK) according to the manufacturer's instructions. Measurements were done using a BMG Labtech CLARIOstar plate‐reader. Data was analyzed using GraphPadPrism software.

### 2.8 Statistical Analysis

Statistical analyses were performed using Excel and GraphPad Prism software. The significance of differences of the means among the groups was assessed by a *T*‐test (two tailed) or by ANOVA (one way) followed by Tukey's multiple comparisons test. The data are expressed as the means ± standard deviation.

## 3 Results

### 3.1 Cell Viability and Proliferation

**FIGURE 1:**
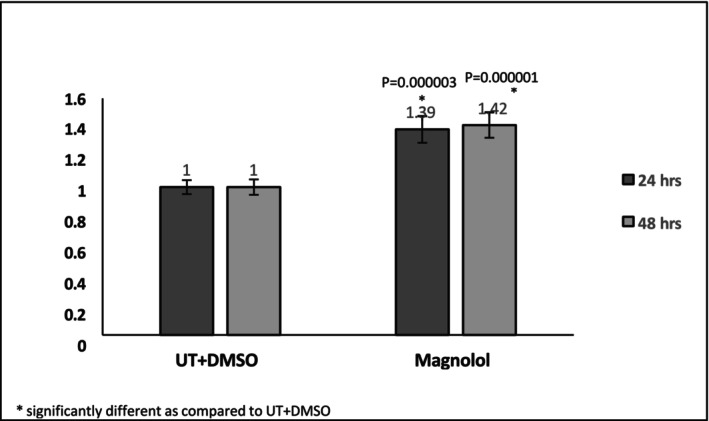
Viability ratio compared to untreated cells and cells with DMSO at 24 and 48 h of incubation; Cell proliferation increased in the presence of ML after 48 h of incubation as compared to the untreated cells and the cells that were incubated with DMSO at the same concentration used to dissolve ML. (UT = untreated).

At 3.16 μM ML‐treated pre‐adipocytes demonstrated a viability of 85.7% and 92.8% at 24 and 48 h, respectively. The ratio of the viability of the ML‐treated revealed a significant increase compared to the untreated cells, suggesting an increase in proliferation (Figure 1). The ratio of the proliferation rates revealed that the cells treated with ML for 48 h demonstrated an increased proliferative capacity as seen in the Figure S1.

### 3.2 Differentiation Assessments

**FIGURE 2:**
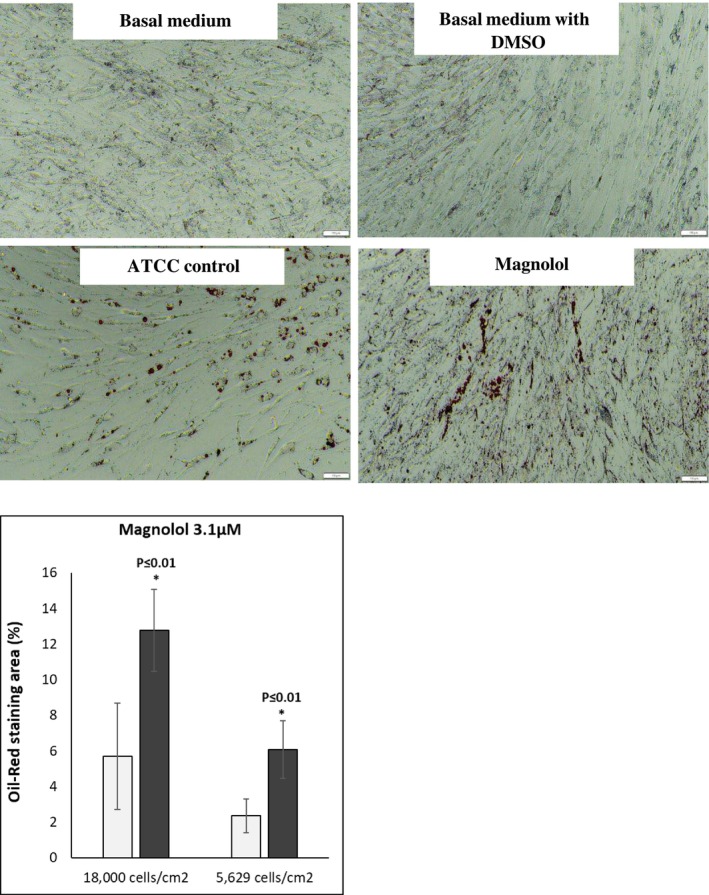
(a) Oil red staining ML versus controls demonstrating pronounced staining of lipid components with the addition of Magnolol (ML). (b) Quantification of oil red staining with different cell numbers showing significant increase adding Magnolol (ML).

**FIGURE 3:**
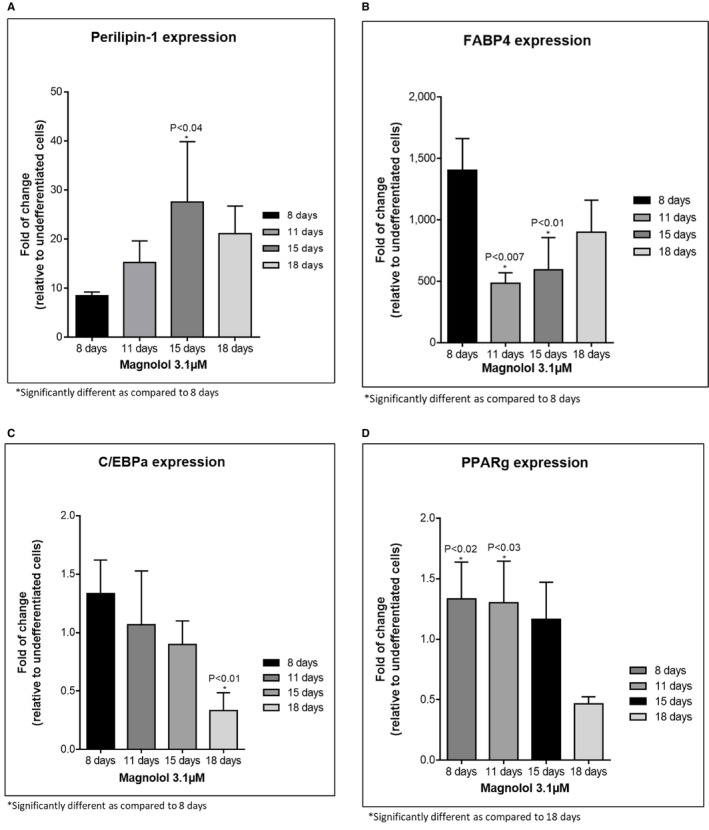
(A) PLIN expression in the presence of ML and control (27.6‐fold increase day 15). *N* = 3. (B) FABP4 expression in the presence of ML and control (1402‐fold increase day 8). *N* = 3. (C) C/EBPα expression in the presence of ML and with control. *n* = 3. (D) PPARγ expression in the presence of ML and with control. *n* = 3.

**FIGURE 4:**
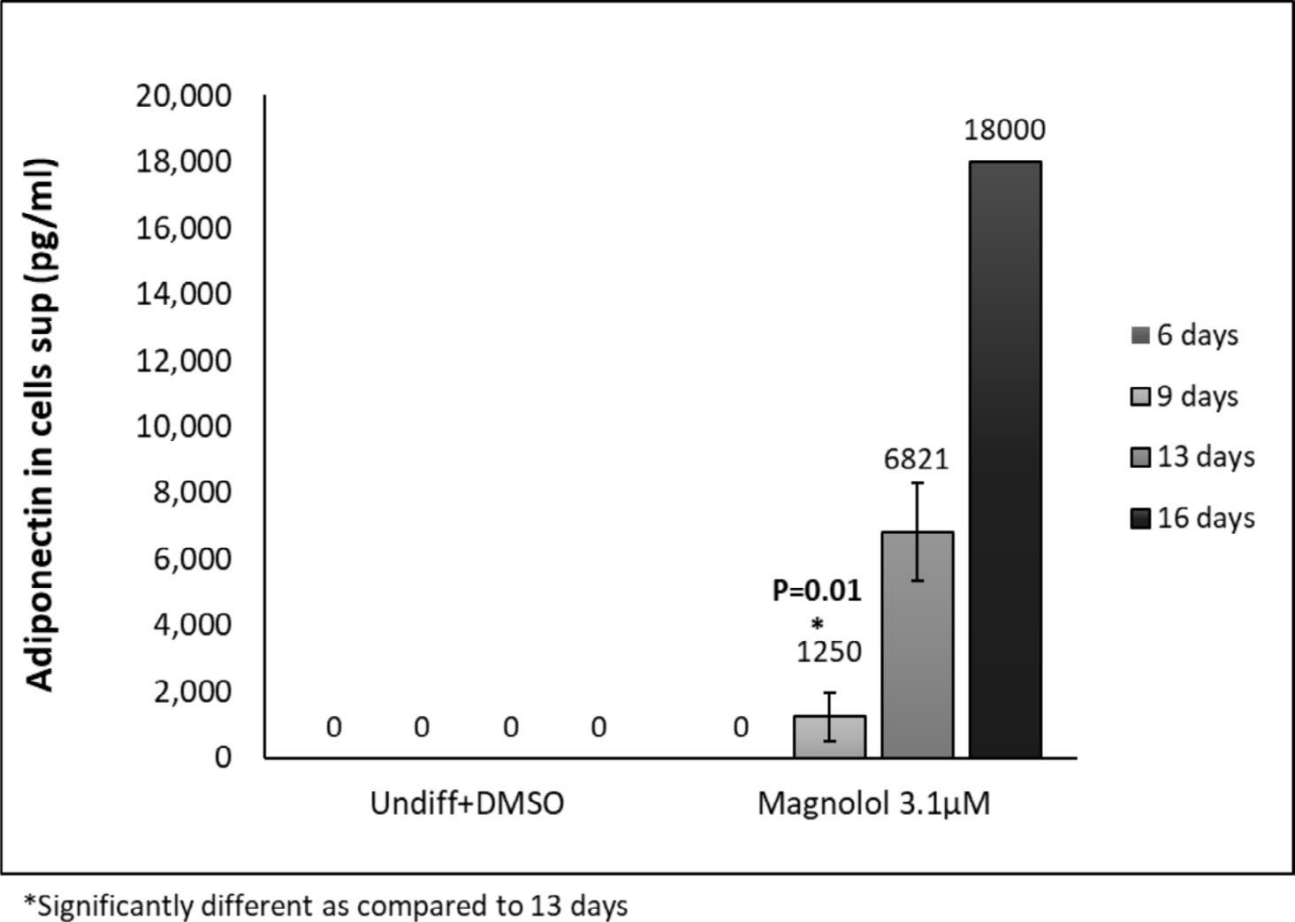
Adiponectin secretion in the presence of ML and with control. *n* = 3.

Oil red staining revealed enhanced lipid‐filled cells when pre‐adipocytes were treated with positive control. Furthermore, treatment with ML induced lipid‐filled cells (Figure 2a,b, oil red staining). Adipogenesis gene expression changes revealed that ML induced the highest gene expression changes at days 9,13 and 16 of differentiation: Perilipin‐1 (PLIN‐45‐fold at day 13); (Figure 3A) d adipocyte‐Type Fatty Acid‐Binding Protein (FABP4–1276‐fold at day 16): (Figure 3B) CCAAT/enhancer binding protein (C/EBP) and peroxisome proliferator‐activated receptor γ (PPAR‐γ) *showed increases at days 9 and 13 but less fold change* than for Perilipin‐1 and FABP4 Figure 3C,D. Finally, the secretion of adiponectin revealed a time‐dependent increase for the ML‐treated cells. (Figure 4).

## 4 Discussion

**FIGURE 5:**
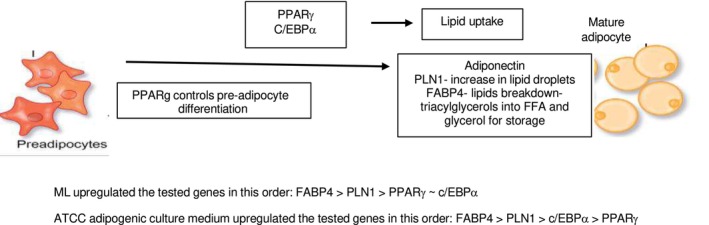
Magnolol stimulates differentiation of pre‐adipocytes to adipocytes primarily through the action of FABP4 and PLN1 with significant increases in adiponectin secretion.

Adipogenesis is the process by which pre‐adipocytes differentiate to mature adipocytes and accumulate as adipose tissue within and beneath the dermis. Adipogenesis can be divided into two steps: the recruitment and proliferation of adipocyte precursor cells, called preadipocytes, followed by the subsequent conversion of preadipocytes, or differentiation, into mature fat cells [6]. The differentiation of pre‐adipocytes to adipocytes involves dramatic alterations in cell shape as well as molecular changes that lead to increase in the ability of the cell to synthesize lipids and secrete adipokines [7]. The proliferation and differentiation of adipocytes involve complex processes controlled by multiple genes, in which PPARγ is the main regulator aided C/EBPa. This initiates the adipogenesis pathway with downstream secondary genes such as FABP4 then being stimulated generating fat cells that secrete PLN1 and adipokines such as adiponectin (Figures 4 and 5).

Polyphenols, a group of natural compounds, are known for their anti‐oxidant and anti‐inflammatory activity. When comes to skin related adipogenesis, varied effects on adipogenesis have been reported. White tea extract, curcumin, epigallocatechin gallate (EGCG) the primary green tea catechin, resveratrol and soybean polyphenols all demonstrated suppression of adipogenesis under various conditions [8, 9, 10, 11]. Moreover, due to their immense diversity and depending on their source and purity, polyphenols can act in various ways related to adipogenesis, both stimulatory and inhibitory. In our study, using pure (98%–100%) ML on normal primary human pre‐adipocytes that were derived from a healthy 33 year old Caucasian female (ATCC PCS‐210‐010), we found ML demonstrated a clear induction of adipogenesis.

In this study, ML demonstrated excellent activity in maintaining viability and accelerating proliferation of the pre‐adipocytes. Preadipocyte replication and differentiation decline with age at various rates and among different body parts.

Another critical factor seems to be variability in sun‐exposed skin. Kruglikov IL and Scherer PE suggest that chronically photo‐damaged skin displays a fibrosis of dermal and subdermal adipocytes, in a process described a “adipocyte‐myofibroblast transition” (AMT), with myofibroblasts being the primary fibrogenic effector cells [4]. There are strong indications that the transition of adipocytes to mesenchymal cells substantially contributes to the development of cutaneous fibrosis. It may be an important part of extrinsic skin aging, whereby both the reduction of dWAT and sWAT and substitution with fibrotic structures are contributing factors [4]. PLN1 is also known as a lipid droplet associated protein that is capable of anchoring itself into the membranes of lipid droplets.

FABP4 is a lipid transporter that is a target gene for PPAR‐γ. PPAR‐γ induces FABP4 almost exclusively. Our study demonstrated that, especially after 16 h of incubation with ML, FABP4 was significantly upregulated, while PPAR‐γ was decreased but this was not statistically significant. This finding is in line with other reported observations demonstrating that increased FABP4 is associated with a potential decrease in PPAR‐γ. The mechanism for these inverse relationships is explained by the fact that FABP4 triggers the ubiquitination and subsequent proteasomal degradation of PPAR‐γ [12]. Therefore, the upregulation of FABP4 in subcutaneous‐derived pre‐adipocytes by ML is meaningful.

One of the most significant findings in this study is the dramatic increase in adiponectin secretion induced by ML. In fact, after 9 days of incubation with ML, the secretion of adiponectin increased by 2 orders of magnitude when compared to the adipogenic medium, which served as a positive control: 18000 versus 9047 pg/mL respectively. The expression is significantly increased at day 13 as compared to day 9. The difference is not statistically significant at earlier time‐points since adiponectin expression is only increased when the cells initiate differentiation.

Adiponectin, an adipokine secreted by adipocytes regulates homeostasis of glucose levels, lipid metabolism, and insulin sensitivity. This is done via its anti‐inflammatory, anti‐ fibrotic, and antioxidant effects. In adipocytes, adiponectin affects the consumption of glucose, increase in triglycerides and fat storage [13]. Importantly, it is becoming well recognized that adiponectin has significant effects on skin health—improving skin barrier homeostasis, increasing ceramide levels, anti‐photoaging effects through downregulation of p38, increasing hyaluronic acid (HA) production, pigmentation control, promoting M2 macrophage polarization, and suppressing TNFα activity [14]. As such, it is considered one of the most important adipokines released with healthy adipogenic stimulation.

While the increased accumulation of fat in adipocytes is expected to generate a certain amount of volumizing/plumping effect in the skin, ML, through its upregulation of adiponectin expression, more importantly, should improve skin health and may also exert anti‐fibrotic effect associated with skin aging. Interestingly, when ML was used at 31.6 μM at one time exposure to activated pre‐adipocytes, we observed enhanced cell differentiation with less cellular proliferation. However, at 10 times lower concentration (3.16 μM), ML significantly facilitated differentiation but did not inhibit proliferation and perhaps even accelerated it. This was in contradiction to the positive control, which demonstrated accelerated differentiation but halted proliferation. It is well known that both cell differentiation as well as cell proliferation are slowed in aging and more cells enter into senescence where they no longer proliferate. As such, this dual activity of ML may be unique and serve to address both processes. This unexpected finding may be of great importance as an anti‐aging benefit by increasing cellular differentiation to create new healthy fat cells while increasing their proliferation. Small healthy fat cells are the sought after regenerative response—the initiation of hyperplasia rather than hypertrophy of the adipose cells.

## 5 Summary and Conclusions

Magnolol was chosen after screening 12 actives for their cytotoxicity/viability and proliferative effects on pre‐ adipocytes (data not shown). ML was selected due to its ability to maintain cell viability and proliferation.

Our results demonstrate the following:

- Significant increase in preadipocyte metabolism after 24 and 48 h of cell incubation with ML at 3.16 μM (Figures 1 and 2)
- Suggested increase in proliferation rate after 48 h of incubation (Figure 2)
- Significant accumulation of fat in adipocytes as demonstrated by oil red staining (Figure 2a,b)
- Increased gene expression of PLN1 and FABP4 (Figure 3A,B) These values were statistically significant with a fold change above 2.
- Dramatic and significant increase in adiponectin protein expression (Figure 4)

Adipose tissue was historically thought of as merely a fat storage organ; however, it is now known to act as an active endocrine organ, which secretes various types of bioactive molecules, including leptin and adiponectin (APN) [1, 2, 3, 4].

This provides opportunities to address skin aging via a novel approach that targets adipose tissue in dermal and subcutaneous planes possibly diverting adipocyte phenotypes from senescent and inflammatory nature to those stimulating regenerative adipokines with positive effects on skin health and esthetics.

## Author Contributions

A.D.W. and N.D., developed the science, data analysis, paper writing. M.E.Z., developed science and paper writing. F.S., paper writing, data analysis.

## Conflicts of Interest

Widgerow is Chief Scientific Officer of Galderma. Shafiq is full time employee of Alastin Skincare. Dayan and Ziegler are consultants to Alastin Skincare.

## Supporting information


Data S1.

## Data Availability

The data that support the findings of this study are available from the corresponding author upon reasonable request.
