# Supplementary material for: Adipogenic Effect of Magnolol in Primary Human Pre‐Adipocytes With Potential Skin Health and Volumizing Effect
Source: J Cosmet Dermatol. 2025 Mar 26;24(4):e70066. doi: 10.1111/jocd.70066 (PMC11938804; doi:10.1111/jocd.70066)
Supplement: Supplementary file 1 — Data S1. [file JOCD-24-e70066-s001.docx]

**Supplementary Material**

***Table 1****: Set of IDT primer sets*

| **IDT Primers** | **Sequence** | **Tm (°C)** | **Ref #** |
| --- | --- | --- | --- |
| ACTB P2 | ACAGAGCCTCGCCTTTG | 55.2 | 236040273 |
| ACTB P1 | CCTTGCACATGCCGGAG | 56.9 | 236040272 |
| RPL13α rev | TACTTCCAGCCAACCTCGTGAG | 58.7 | 235742811 |
| RPL13α for | CTCAAGGTGTTTGACGGCATCC | 58.5 | 235742812 |
| Perilipin-1 rev | AGACTTCTGGGCTTGCTGGTGT | 61 | 235742821 |
| Perilipin-1 for | GCGGAATTTGCTGCCAACACTC | 59.7 | 235742822 |
| FABP4 rev | GCGAACTTCAGTCCAGGTCAAC | 58.2 | 235742819 |
| FABP4 for | ACGAGAGGATGATAAACTGGTGG | 56.3 | 235742820 |
| C/EBPα rev | AGTGCGCGATCTGGAACTGCAG | 62.1 | 235742817 |
| C/EBPα for | AGGAGGATGAAGCCAAGCAGCT | 61 | 235742818 |
| PPARγ rev | GGCTTCACATTCAGCAAACCTGG | 59 | 235742815 |
| PPARγ for | AGCCTGCGAAAGCCTTTTGGTG | 60.9 | 235742816 |

***Figure S1:*** *Proliferation ratio compared to untreated cells and cells with DMSO at 24 and 48 hours of incubation.*
